# Adipose Stem Cell‐Derived Exosomes for Lichen Planopilaris: A Case Report

**DOI:** 10.1111/jocd.70333

**Published:** 2025-07-05

**Authors:** Firas F. Haddad, Serena Saade, Rami Abadi

**Affiliations:** ^1^ Faculty of Medicine American University of Beirut Beirut Lebanon; ^2^ RA Clinics Beirut Lebanon

**Keywords:** hair, hair disease, hair disorders

To the editor,

A 35‐year‐old female patient presented to our clinic with a 4‐year history of redness, itching, and hair loss over the vertex of the scalp. On physical exam, a scaly erythematous patch of hair loss was observed over the vertex, with atrophy and scarring of the surrounding tissue. A 4 mm punch biopsy (Figure 1) revealed an acanthotic epidermis, with focal and discrete basal layer vacuolization and widespread dermal fibrosis. Within the dermis, there were two atrophic hair follicles surrounded by a concentric fibrous tissue and a lymphocytic exudate. Findings were consistent with lichen planopilaris (LPP).

**FIGURE 1:**
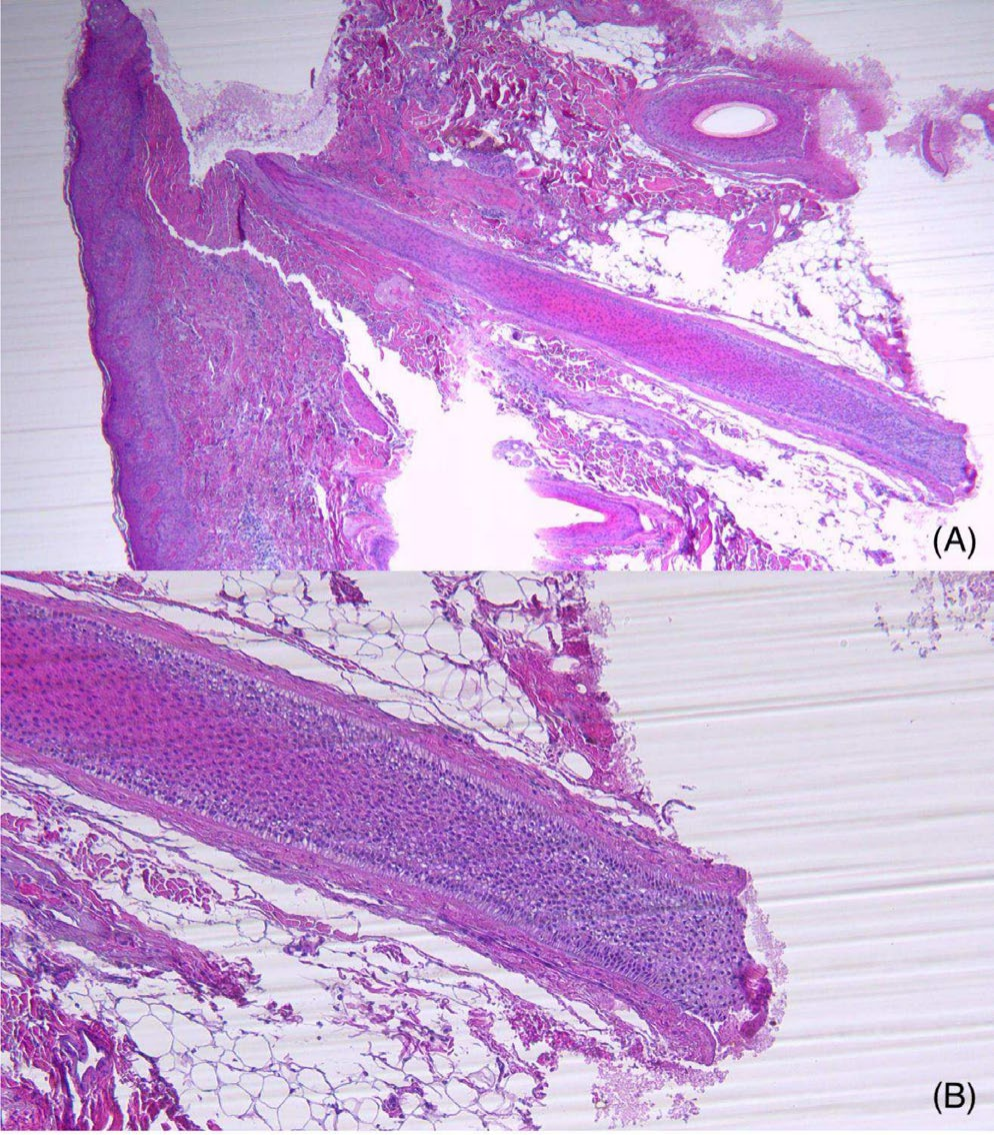
(A) Low‐power view (4X) showing acanthotic and hyperkeratotic epidermis with focal basal layer vacuolization and widespread dermal fibrosis. (B) (10X) High‐power view demonstrating atrophic hair follicles surrounded by concentric fibrous tissue (perifollicular fibrosis) and lymphocytic infiltrate, characteristic of lichen planopilaris.

After declining systemic medications and reporting poor compliance with topical steroid creams due to the greasy appearance on her hair, the patient received three consecutive sessions of intralesional triamcinolone (10 mg/mL, 1.5 cc total volume, injected at 1 cm intervals). Despite treatment, she reported persistent erythema, further scalp atrophy, and no hair regrowth, which prompted consideration of alternative treatment options.

Exosomes derived from human adipose tissue‐derived mesenchymal stem cells were discussed, using ASC‐Exosomes (ASCE+HRLVE, 20 mg; ExoCoBio Inc., Seoul, South Korea). The treatment protocol involved reconstituting freeze‐dried exosome powder with 3 mL of normal saline. After cleansing the scalp with alcohol, microneedling was used to create micropores at a depth of 0.5 mm to minimize the independent effects of microneedling. The exosome solution was then applied topically to the treated areas. The patient underwent four sessions at 4‐week intervals.

Following treatment, the patient showed significant hair regrowth, decreased erythema, and improvement in scalp scarring (Figure 2). Additionally, she reported reduction in inflammation and pruritus.

**FIGURE 2:**
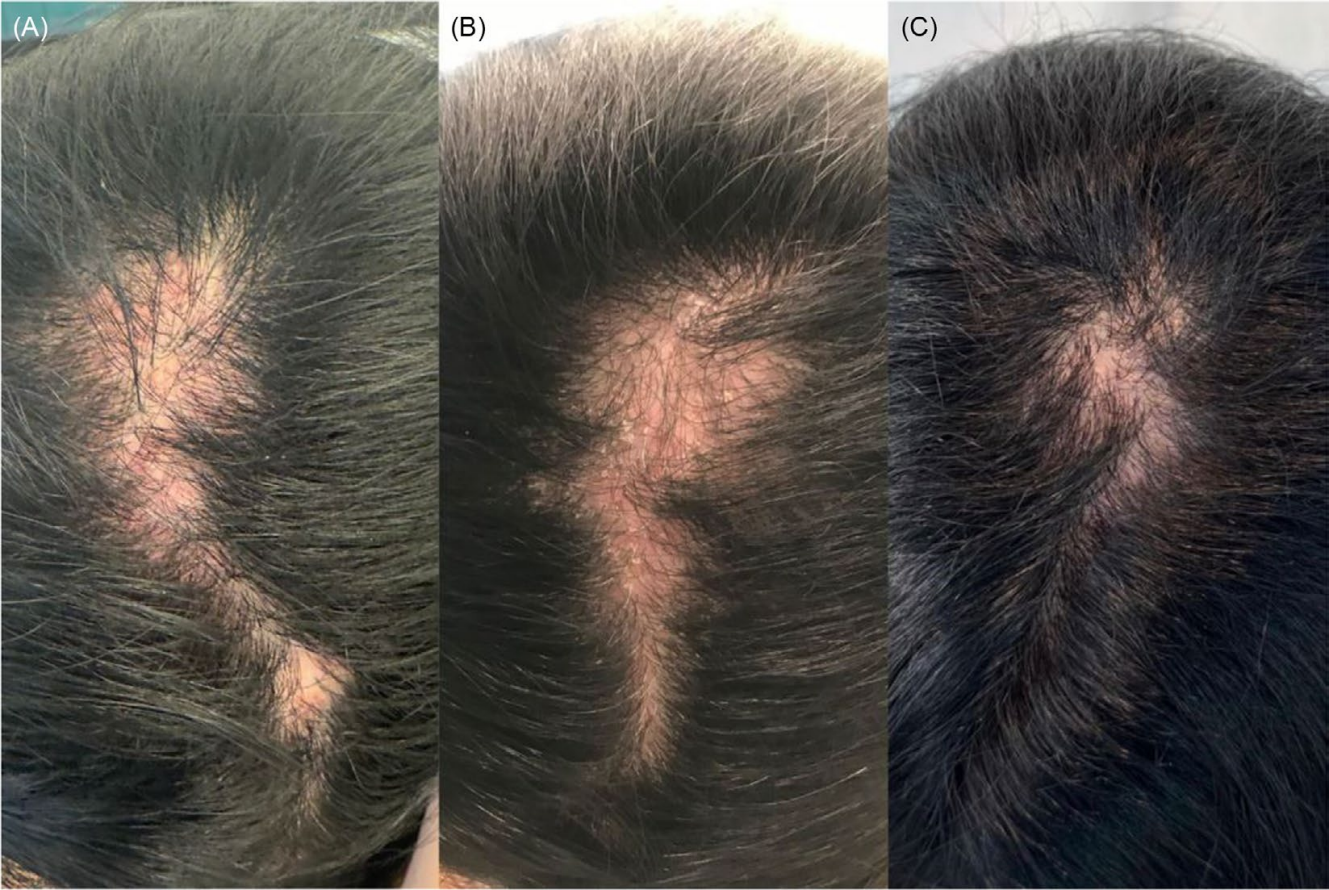
(A) 35‐year‐old female patient. (A) At baseline (B) After an unsuccessful treatment with three consecutive sessions of intralesional triamcinolone (10 mg/mL, 1.5 cc total volume, injected at 1 cm intervals). (C) 16 weeks after the initiation of treatment with exosomes from adipose‐derived stem cell.

LPP is a primary lymphocytic scarring alopecia, characterized by patchy hair loss of the vertex and/or parietal scalp with associated erythema, pruritus, scalp atrophy, and scarring [1]. The pathophysiology likely involves lymphocyte‐mediated autoimmune destruction of hair follicles [1]. Treatment options include topical or intralesional corticosteroids and systemic medications such as doxycycline or hydroxychloroquine [1]. Although intralesional steroids are among the first‐line agents, patients often have a lack of response warranting other treatment methodologies. Less studied options include methotrexate, JAK inhibitors, mycophenolate mofetil, cyclosporine, and platelet‐rich plasma.

To our knowledge, this is the first report of successful LPP treatment with exosomes. Exosomes are cell‐derived vesicles containing carbohydrates, proteins, lipids, and nucleic acids that communicate with neighboring cells [2]. These vesicles modulate important cellular activities at the skin level and have been studied in several dermatological conditions such as psoriasis, atopic dermatitis, vitiligo, systemic lupus erythematosus, systemic sclerosis, and diabetic wound healing [2]. They promote hair regrowth by potentially antagonizing dihydrotestosterone effects at the hair follicle [3] and promoting telogen‐to‐anagen phase transition [4]. However, their role in attenuating autoimmune activity is less understood; however, they have been documented to be effective in other autoimmune forms of hair loss, such as alopecia areata, has been documented [5]. The mechanism likely involves immunomodulation, anti‐inflammatory effects, and tissue repair and regeneration.

Limitations of this case report include the lack of long‐term safety and efficacy data. To add, we acknowledge the limitation of a single case in reaching definitive conclusions regarding the use of exosomes in LPP. Other limitations include the absence of post‐treatment histopathological confirmation. Larger studies are needed to assess the role of ASC‐Exosomes in LPP treatment. The follow‐up period was relatively short, and the optimal number of treatment sessions remains to be determined. Prospective controlled studies with larger patient cohorts are needed, with longer follow‐up periods and the integration of trichoscopy, dermoscopy, and histopathological assessments.

Nevertheless, exosomes offer a steroid‐sparing option with a favorable short‐term side effect profile. Additionally, their potential benefits in wound healing may prove valuable in managing both hair loss and scalp scarring.

## Conflicts of Interest

The authors declare no conflicts of interest.

## Data Availability

Data sharing is not applicable to this article as no new data were created or analyzed in this study.
